# The Diagnosis, Classification and Nature of Tumors

**Published:** 1888-04

**Authors:** Henry F. Formad

**Affiliations:** Philadelphia


### ART. XIII.—THE DIAGNOSIS, CLASSIFICATION AND NATURE OF TUMORS.

BY HENRY F. FORMAD, B. M., M. D., PHILADELPHIA.

### AND AN ACCOUNT OF TUMORS IN DOMESTIC ANIMALS FROM THE CLINIC OF THE VETERINARY DEPARTMENT OF THE UNIVERSITY OF PENNSYLVANIA.

BY R. S. HUIDEKOPER, M. D., VETERINARIAN.

Any swelling that has arisen upon the body without evident symptoms of inflammation which is permanent and has a tendency to increase, is traditionally designated as a tumor.

The terms "tumor," "neoplasm," "pseudo plasm," "new formation," "new growth," and "swelling," are synonymously and indiscriminately used when speaking of tumors.

The nomenclature of tumors, generally adopted, is based upon their histological characters, except in the case of sarcoma (fleshy tumor), and cancer or carcinoma (crab-like tumor), which, however, may be called "endothelioma" and "epithelioma," in order to match them with "fibroma," "neuroma," "osteoma," and all the rest of the tumors in which the name at once designates their morphology.

Every tissue in the body appears to be represented in tumor formation, and there is no tumor made up of elements foreign to physiological life. The histological elements composing tumors differ, however, in some essential features from those of normal tissues. The cells are, as a rule, larger (except in the case of muscular tissues), they are less uniform in size and shape, the intracellular network of the protoplasm of the cell is coarser and less regular, and above all, there is not that definite arrangement of fibres into bundles, and not that fixed and regular uniform relation between cells and fibres (in the case of tumors) that we find in normal structures and tissues.

Pathological cells breed much faster than normal ones, have as a rule, larger nuclei, and often two or more nucleoli. Tumors form an intrinsic part of the individual upon whom they grow, and usually are composed of a tissue allied more or less to that in which they grow, representing nearly a superfluous mass of tissue quantatively above physiological necessity and limits.

Qualitatively, even when perfectly homologous (*i. e.*, fully representing the tissue in which they grow, they differ essentially from normal tissue masses in not being endowed with any physiological function, and this on account of the absence of all the essential regulators of life, such as nerves, lymphatics and organized blood-vessels. The blood does circulate through the tumor, but not through arteries and veins, but, as a rule, through systemless channels which have no vaso-motor power on account of absence of muscular walls and absence of regulating nerves. It is on this account that tumors are painless in themselves unless mechanically pressing upon nerves, with the exception of neuroma, which represents nerve tissue in itself, and the cancers which are strictly no new formations "in masse," but represent a pre-existing connective tissue, the lymph spaces of which are stuffed with foreign intruding epithelial cells, which, like any other foreign mass, presses upon the nerves contained in the pre-existing tissue.

Tumors are not identical with hypertrophies. Tumors are true hyperplasias, *i. e.*, their bulk is due to multiplication of tissue elements and not to mere enlargements of the elements, as is the case in true hypertrophies. Furthermore, hypertrophied parts, for instance, a hypertrophied heart or a hypertrophied gland, show rather an increase in functional activity, whereas tumor masses have no function at all. Just as striking appears the distinction between purely inflammatory new formations and tumors. Whereas the latter may represent most any tissue morphologically, most any tissue in creation, as is seen in the various tumors, the inflammatory process in itself can, strictly speaking, give no other results than the formation of pus and destructive disturbances in the tissues in the case of acute lesions and in the formation of connective tissue which likewise impairs tissue elements through pressure in which it grows in the case of chronic lesions.

Yet I am strongly inclined to regard tumors as one of the terminations of inflammation, as it is often very difficult to establish where the inflammatory process ends and where the tumor formation begins. The result of my studies in this direction which I carried on quite diligently some years ago can be found in the transactions of the Pathological Society of Philadelphia for 1881, and in some of the medical journals of that time.

Nowhere is the etiological relation between the inflammatory process and tumor formation so close and evident as in comparative pathology as will be seen in the account of tumors in domestic animals to be recorded below from the cases of Professors Huidekoper, Zuill and other veterinarians.

The difficulty in connecting more closely the etiological relations of inflammatory formations and tumors, lays in the fact that a number of congenital anomalies or malformations are classified with tumors. I have reference to Angioma, Lymphangioma, Dermoid tumors, Kelloids and other Naevi, Rhabdo-myoma and certain forms of adenoma and cysts.

All these new formations are surely congenital, and could surely better be classed with the monstrosities or false tumors. I consider the following neoplasms which are composed of new-formed or overgrown tissues as *true* tumors: Fibroma, Lipoma, Chondroma, Osteoma, Leio-myoma, Myxoma, Lymphoma, Sarcoma in all its forms, Glioma, Actinomykoma, Papilloma, Carcinoma, Tyroma (tubercular tumor), Gumma, Lupus; simple Epithelioma (as represented by, corns, horns, onychoma, etc.)

All of these tumors have been traced in various instances directly or indirectly to traumatism and various other forms of irritation.

Cohnheim was, as is well known, the originator of the "congenital," or embryonal theory of tumor transformation. He uses the well-established congenital derivation of the dermoids, rhabdo-myoma, angeioma, etc., as a basis for his hypothesis, and jumps at once to the conclusion that all tumors are congenital and of embryonal origin. Some new formations which he admits to be of inflammatory origin —viz., gumma, tubercle, lupus, neuroma, osteophytes, etc. —he excludes from the category of true tumors.

But how great a reduction in number will Prof. Cohnheim's list of true tumors experience should it be proven that all tumors are of inflammatory origin save those few congenital formations which I suggest to exclude from the list of true tumors.

In the cases of tumors in which I had the opportunity to get the history of myself, or where I insisted upon an exhaustive anamnesis in cases of others, it was possible in nearly one-half of the cases to trace out a local inflammatory process preceding the tumors at some time or other. Sometimes it dated years back. Careful inquiries nearly always revealed some cause,—viz., an injury, long standing irritation, mechanical or toxic, or an impaired or excessive use of the part, pressure, or a long-standing catarrh, or something of that nature.

It is also an established fact that those organs and regions of the body which, from their position and their function, are most exposed to injuries or irritation are the most usual seat of tumors. This is proven for the orifices of the digestive and genito-urinary tract, which are so much exposed to injuries and are also classical seats of especially malignant tumors.

Primary cancer of gall-bladder has been repeatedly traced to gall stones; that of the urinary bladder to a similar cause. For surface-cancers an inflammatory origin may safely be regarded as proven. I know of scores of epitheliomata which had been traced to little sores produced by injury. Nearly all those everlasting leg ulcers are epitheliomata.

It is just here that the influence of evolution and involution of tissue upon the variety of tumor does not hold good. Repeatedly have I seen epitheliomata of lower extremities in young persons directly produced by injuries, burns, etc.

Who will deny the inflammatory origin of the very common epithelioma of lip? or that of tongue or penis?—the first nearly exclusively occurring in smokers, the second always being associated with injury of tongue by sharp teeth or otherwise, and the third with congenital or acquired phimosis. Chronic inflammations of the skin, as occurring on workers in coal-tar and paraffin manufactories, etc., commonly lead to epithelioma. A similar origin has the chimney-sweeper's cancer. It has been proven that long-continued catarrhs of stomach (particularly in drunkards) lead to cancer. Through irritation and injury common warts and scars are produced; further repeated injuries very frequently convert them into malignant tumors (cancers, sarcomata).

Prof. Agnew removed from the back of a middle-aged person a sarcoma developed secondarily in a scar. Some time previously he had removed (from the same patient) from the same spot, or from above that spot, a lipoma. Sarcomata are commonly due to direct injury; neuromata exclusively so. Glioma and tyroma, as met with in the brain, are nearly always traceable to falls and blows. Hundreds of cases of fibroma, lipoma, chondroma, and osteoma have been traced by distinct and clear histories to pressure and irritation, or directly to blows, fractures, cuts, and other injuries.

Winkel, who investigated exhaustively the etiology of fibromata and myomata of the uterus, came to the conclusion that these tumors are caused either by direct excitants, viz.: coition, injury, abortion, rough removal of placenta, cellulitis, or, indirectly, through repeated lifting, shock, sudden hyperæmia, etc.

No reliable line of distinction can be drawn between the lymphomata and lymphadenitis.

Any one can convince himself of the above-mentioned facts by just looking carefully over the literature, and by taking careful histories of his own cases. Hundreds of tumor cases of positively traumatic origin are also recorded in the classical works on tumors of Virchow, Weber, Müller and Broca.

Tissues which are most liable to be the seat of inflammation are also the most *common seat* of tumors. Again, those tissues which do not participate in active inflammatory processes (ganglionic and striated muscular tissue) seldom or never give rise to tumors.

The extensive and careful statistics of Dr. D'Espine, of Geneva, show that the os uteri and the stomach are the most frequent seats of primary cancer, and that they are also distinguished for their remarkable liability to catarrhs. Virchow has repeatedly pointed out in a catarrhally inflamed gastric mucous membrane the gradual transition to carcinoma, a fact observed also by Dr. J. H. Musser and myself.

The healing process in malignant tumors (wherever it occurs) is precisely the same as that of an ordinary granulating ulcer. Here and there, healing is accomplished by the additional formation of connective tissue,—*i. e.*, cicatrization.

But the most beautiful analogy between tumors and inflammatory products is demonstrable by the microscope.

### NOMENCLATURE.

I will not enter here into the minute consideration of the structure of tumors. It is sufficient to recall that the technical name of each tumor indicates its structural composition, so the term fibroma indicates nothing else but a tumor made up chiefly of connective (fibrous) tissue ; Neuroma, one of nervous tissue, and so on. When two or more tissues enter into the histological composition of a tumor, then the names are combined, *e. g.:* Fibro-myoma, Myxo, Chondro, Sarcoma, etc. When a tumor has undergone cystic change, then the denomination is prefixed by the term "cysto," such as, Cysto-Sarcoma, Cysto-Adenoma, etc.

### CLASSIFICATION OF TUMORS BY NATURE—BENIGNITY, MALIGNITY.

---

The following table will be found of practical use in Clinical Diagnosis :

### DIFFERENCE BETWEEN TUMORS.

| *Benign.* | *Malignant.* |
|---|---|
| 1. Are homologous and typical. | 1. Are heterologous and atypical. |
| 2. Present a perfect tissue, rich in fibres and poor in cells—cells large. | 2. Present an imperfect tissue ; poor in fibres and rich in cells—cells small. |
| 3. Grow centrally. | 3. Grow peripherally. |
| 4. Have a capsule. | 4. Are not encapsulated. |
| 5. Usually poor in blood-vessels. | 5. Are rich in blood-vessels. |
| 6. Usually firm in consistence and dry. | 6. Usually soft or juicy. |
| 7. Seldom ulcerate. | 7. Often ulcerate. |
| 8. May grow large. | 8. Seldom grow large. |
| 9. May be primarily multiple. | 9. Never primarily multiple (except melanotic sarcoma). |
| 10. Do not re-occur after removal. | 10. Do re-occur. |
| 11. Give no metastasis. | 11. May give metastasis. |
| 12. No cachexia. | 12. Cancer alone gives rise to cachexia when metastasis has occurred. |
| 13. May kill by mechanical pressure and hemorrhage. | 13. Killed by destruction of tissue and metastasis. |

Metastasis, or the deposition of secondary foci from a primary malignant tumor to other parts in the body, usually, if not always, takes place only to dissimilar structures and always from outer to inner, or deeper structures. Metastasis to the skin does not occur, so that when there are a number of peripheral tumors, they are all independent and primarily multiple. Multiple peripheral tumors are as a rule benign, except the melanotic sarcoma.

Some tumors are malignant only locally, *i. e.*, they re-occur after extirpation, and while producing great local tissue destruction, they do not give metastasis on account of the large size or length of the cells composing them. Here belong in particular, the Squamous Epithelioma, the giant-celled, large-celled and spindle-celled Sarcomata.

It is not the want of resistance of the surrounding tissue

(as is generally held), but simply the getting loose of the normal cells from their place of attachment, which constitutes the formation of a malignant tumor.

It is the mobility and small size and round shape of the cells, I think, that conditions the malignancy of a tumor. Any tumor, even the most benign one, would be malignant if the cells composing it could get loose and travel through the widely open paths of the system of juice-channels and blood-vessels. In benign tumors the cells are more or less fixed, hence no metastasis.

In the following table all Tumors known are given in two columns, Benign and Malignant, the latter in the order of their malignity :

| *Benign.* | *Malignant.* |
|---|---|
| Fibroma, soft (peripheral.) | Sarcoma, melanotic. |
| " hard (deep-seated.) | " myxomatous. |
| Lipoma. | " alveolar. |
| Myxoma. | " small round-celled. |
| Chondroma. | " Lymph—adenoid |
| Osteoma, osteophytes. | " large round-celled. |
| " exostosis. | " small spindle-celled. |
| Osteo-chondroma. | " large spindle-celled. |
| Leio-myoma. | " giant celled, or mysloid. |
| Rhabdo-myoma. | Cholesteatoma. |
| Neuroma, medulated. | Psammoma. |
| " non-medulated. | Glioma. |
| Lymphoma, hard. | Tyroma, Lupus. |
| Onychoma. | Gumma. |
| Odontoma, | Actinomykoma. |
| Angioma. | Carcinoma, soft. |
| Lymph-Angioma. | " hard. |
| Goitre. | Epithelioma, cylindrical. |
| Adenoma. | " squamous. |
| Adenoma, cystic. | Carcinoma, colloid. |
| Papilloma, soft, hard. | |

### DIFFERENTIAL DIAGNOSIS OF SARCOMA FROM CARCINOMA (CANCER.)

| *Sarcoma.* | *Carcinoma.* |
|---|---|
| 1. Composed nearly entirely of cells, imbedded into a homogeneous or reticular matter. | 1. Composed of cells laying free in alveoli or spaces formed by pre-existing often massive connective tissue. |
| 2. Cells endothelial or embryonal or lymphoid. | 2. Cells exclusively epithelial. |
| 3. Blood-vessels without muscular walls, and running free between the cells. | 3. True blood vessels (arteries, veins) and nerves running only through the connective tissue frame-work. |
| 4. No fat within tumor proper. | 4. Fat may be seen within the cancer tissue. |
| 5. Metastasis by blood-vessels. | 5. Metastasis by lymphatics. |
| 6. May affect lymph-glands primarily; but not by metastasis. | 6. Never develops in lymph-glands primarily but often infests them by metastasis. |
| 7. Developes in connective tissues substances deep-seated and grows upwards. | 7. Develops from epithelium, usually peripheral, and grows downwards. |
| 8. Skin not adherent. | 8. Skin adherent frequently. |
| 9. Not painful. | 9. Painful. |
| 10. Occurs in young and well-nourished individuals, chiefly. | 10. Occurs chiefly after middle life. |
| 11. Not hereditary. | 11. Often hereditary |
| 12. Grows rapidly. | 12. Grows slowly. |

Alveolar sarcoma, or endothelial cancer, is considered by some pathologists identical with epithelial cancer or carcinoma. But this is not correct—a distinction must be made. Besides, the general differences above stated, the following distinction can be made microscopically and chemically :

| *Alveolar Sarcoma.* | *Glandular Carcinoma.* |
| --- | --- |
| 1. Cells becoming transparent and nearly dissolve in acetic acid. | 1. Cells not affected by acetic acid or potassium hydrate. |
| 2. Cells vary in size, small and large in the same specimen. | 2. Cells uniformly small. |
| 3. Cells stick to alveolus and are removed only with difficulty. | 3. Cells lay free and are easily removed by brushing. |
| 4. Cells irregular shaped and nuclei large and take staining wells. | 4. Cells regularly shaped, nuclei small, do not stain as well. |
| 5. Alveoli formed of young connective tissue. | 5. Alveoli formed of old dense connective tissue. |

### CLASSIFICATION OF TUMORS BY SHAPE.

(Very convenient for clinical diagnostic purposes.)

A. *In deep-seated structures.*
B. *On the periphery of body.*

A. Forms within the parenchyma of organs :

(1) *Uniform Swelling*, means the transformation of a whole organ into a tumor. To this belong : *Goitre, Lymphoma, Glioma* and *Sarcoma* of certain organs ; kidneys, spleen, glands.

(2) *Nodes Growing Centrally* : means a tumor growing from within, outward, the periphery being the oldest part (like the endogenous growths in plants) : A node develops when the conditions are such that pressure on it is equal from all sides, or if there be no pressure on it anywhere.

(a) Benign Tumors. *Fibroma, Myoma, Lipoma, Chondroma, Osteoma, Adenoma, Neuroma.*

(b) Malignant tumor nodes are all *secondary* metastatic *carcinoma* and *sarcoma* (not the primary).

(3) *Nodes by Infiltration*, or Dissemination of Nodes growing Peripherically. (The youngest part being the outside of the tumor.) Here belong all the malignant tumors—*Sarcomata* and *Cancers.*

(4) *Tuber*, or Partly Protruding Nodes covered by Skin.

They are nodulated because they are composed of many nodes united into one mass. This form is assumed by *Osteoid, Chondioma, Osteoma* and *Chondioma.*

B. Forms of Tumors upon Surface.

(5) *Desquamation*, represented by *Icthyosis*, which means a congenital hypertrophy of the epidermis.

(6) *Tilat Tabular Swelling*, is found when the tumor is subjected to pressure evenly from two opposite sides—is an elevation above the surface whose basic diameter is greater than its height.

(a) *Malignant*, growing by infiltration. Here belong: *Lupus, Squamous Epithelioma* and *Cylindrical Epithelioma.*

(b) Benign or Centrally growing: flat tabular swelling, here belong: *Angioma, Lymphangioma, Colloids* and other *naevi.*

(7) *Hemispherical growths* are formed where the resistance is chiefly on one side. *Fibroma, Sarcoma*, spindle-celled (chiefly), and *Gumma.*

(8) *Fungus*: is a pedunculated growth whose top has become largely developed. It is usually a protrusion of a malignant tumor upon the surface and is not covered by skin like a tuber. Here belong: *Vascular* or *Telangiectatic, Sarcomata* and *Soft Cancers.*

(9) *Polyp* or *Pediculated Growth*, develops when the tumor is located quite peripherally beneath the skin, mucous, or a serous surface, which do not resist, and the tumors bulge and then form pediculated growth. Here belong: *Myxoma, Soft Fibroma, Lipoma, Adenoma* and *Bovine Tubercle*, from serous surface.

(10) *Papilla*: means an outgrowth of epidermis alone forming *Horns, Corns* and *Pointed Condylomata* (which is a syphilitic growth).

(11) *Dendritic* or *Cauliflower* growths are connective tissue vegetations from the papillæ of the skin or mucous membrane covered with epithelium. Here belong: *Papilloma*, growing upwards, and *Epithelioma* if growing both downwards and upwards.

(12) *Cysts* are hollow tumors with fluid contents.

There are six varieties; Retention, Exudation, Extravasation, Softening, Dermoid and Parasitic cyst.

(a) *Retention* cysts are due to obstruction of outlets of glands, with subsequent distension of the glandular structure. Instance: 1st *Sebaceous* or *Atheromatous* cysts; 2d, *Ranula* or *Mucous* cyst found in salivary glands and pancreas; 3d, *Cysts due to obstruction of ducts* of internal organs, (Kidneys, liver, etc).

(b) *Exudation* cysts, due to exudation of serum into previously closed sacs. This embraces all the forms of Hydrocele and Hydrocephalus, Hydrothorax Ascitis, etc. Subdivisions are Ovarian cysts, which at first start as retention cysts and then continue as exudation cysts.

(c) *Extravasation* cysts are due to hemorrhage into solid tissue, the subsequent absorption of the clot and its substitution by serum.

(d) *Softening* cysts are due to the liquifaction of a tumor or an organ by means of mucoid or fatty metamorphosis. Instance: *Cysto-Fibroma* or *Cysto-Carcoma.*

(e) *Dermoid* cysts are congenital invaginations or misplacements of the skin and contain in their interior any structure pertaining to the skin, as hair, teeth, etc.

(f) *Parasitic* cysts are due in man to the embryo of the tapeworm in the dog, the Tenia Ecchinococcus.

### TUMORS OF DOMESTIC ANIMALS.

As to the relative frequency of occurrence of tumors in domestic animals, the following statistics of tumors from the veterinary clinics of the University of Pennsylvania may serve as an index.

The tumors here recorded were shown before the last two meetings of the Keystone Veterinary Medical Association, and they represent, as far as collected, the regular material of the veterinary clinic for about two years. There are not included, however, a large number of the more common tumors that were lost for want of proper preservation.

The tumors are enumerated in the order of their frequency of occurrence, and are divided into two groups (a.) Malignant tumors, and (b.) Benign tumors.

### A. Malignant Tumors.

These are much more common in the domestic animals than they are popularly supposed to be, or than one would infer them to be by reference to any of the veterinary text books, the most common are:

I. Melanotic or Melanotic Sarcomas.—Specimens from eight cases, were all of them subcutaneous, and the tumors had been multiple in each animal. They occur most frequently in the horse, sometimes in the mule. They rarely occur in cattle and in the dog. In horses and the mule they appear almost invariably in grey animals. An old Arab tradition will be found absolutely true; "that a grey horse with curly mane and tail has black tumors." The growth of these tumors is very variable—frequently they exist for a long time only as small subcutaneous tumors, especially on the under surface of the tail and around the softer skin of the anus, or again at other parts of the body. These may frequently disappear by absorption leaving an *unpigmented* spot. In other cases they may grow in a few weeks to the size of a goose egg, or a man's head; the animal occasionally turning from a dark grey to almost a white in that time. By metastasis or primarily the tumor may be located in the lymphatic glands, in the loose connective tissue of the muscular interspaces, or between the scapula—great serratus (one case caused lameness); in the mesentery, causing colic; in the lungs, giving according to the rapidity of their growth, any of the train of symptoms found in other troubles, from a chronic pneumonia and the heaves to an acute pneumonia ending in a pleurisy. In the mediastinum we sometimes find enormous growths, these may brake in the pleural cavity causing a pleuritis, or may remain harmless. In one case a tumor the size of a child's head, evidently of slow growth, had pressed the aorta and pulmonary artery far to the left, had driven the auricles down, and to the left so that the superior surface was concave, and had greatly deformed the right side of the ventricles. This animal was destroyed for other trouble, having worked up to the day of its death without having aroused any suspicion of heart trouble. In one case a small melano sarcoma under the left parotid gland was the cause of a facial paralysis on that side.

Dr. Rayner reported a melanotic tumor of the pelvis weighing thirty-five pounds. The writer had a case where complete occlusion of the rectum had prevented any passage for a week; relief by operation was attempted but severe hemorrhage from the internal pudic necessitated a ligature at a cubits length from the anus, and the animal died on the fourth day of peritonitis. Two cases appeared in the mammary glands of mares—one of rapid growth interfered so with the action of the animal that removal of the glands was necessary, the other was of slow growth. These of course destroyed the value of the animals entirely as they were intended for breeding.

Melanotic tumors will sometimes disappear spontaneously, and not recur in several years observation.

II. Epithelioma Squamosa.—These growths are found most frequently on the glans penis of the horse, and may encroach upon the entire free portion of the penis and even extend to the sheath. There is a constant tendency for them to enlarge, and they are frequently found to recur after removal. Again, in the horse they may appear at any part of the body and in two cases were multiple. A sorrel gelding, 10 years old, 15½ hands, belonging to Mr. D——, was used for light delivery work. Had developed an epithelioma two years previously, on the right superior maxilla which had been removed. In six months it returned, accompanied by similar growths under the abdomen, at a later period on the side of the neck. In the course of a year the writer operated some four or five times removing the maxillary tumor twice, and the others as they incommoded the use of the animal. Some of the cicatrices remained sound while the tumors reappeared in others and in new localities, until the animal had to be destroyed. Epitheliomas are found on the tail of the horse apparently the result of injury or irritation. The irritation of a foul penis and sheath, is frequently the cause of the growth at this point. The nose and edges of the lips in the dog and cat, is a common seat of the growth in these animals. It is seen again on the paws of the dog. (1 case.)

III. GLANDULAR CARCINOMA.—This growth we have found only in the dog (7 cases.) Five of these were mammary, one of them in a small terrier bitch weighing only about ten pounds without the tumor, which weighed over a pound and hung down to the ground when the animal was standing. At the posterior extremity, reaching nearly to the vulva, was a fatty growth of some size and on the dependent portion, was a well developed papilloma the size of a half dollar. Another of the mammary carcinomas was colloid. The sixth case was in the stomach of a dog, while the last developed in the pancreas and in the liver.

IV. MYELOID OR GIANT-CELLED SARCOMA.—Several cases in cattle occurred in the lower jaw, and at their origin may frequently give rise to suspicion of actinomycosis. Two cases in the superior maxilla of the horse were of extremely rapid growth, distorting the sinuses, and in one case completely forcing the eye from its socket. Another case had its primary development on the tail, enlarging that organ to enormous size and rapidly traveling to the root. The animal was destroyed and at the autopsy multiple cancers were found in the liver, the spleen, the lungs and mesentery.

V. SPINDLE-CELLED AND ROUND-CELLED SARCOMAS of the horse have been found less frequently than the last and were all located subcutaneously.

VI. LUPUS in the multiple tumors was found in one case on the wing of a young fowl (8 months), the remainder of the flock were examined, but were found sound.*

B. Benign Tumors.

I. Cysts.—These we find in large numbers in all of the domestic animals. *Ovarian cysts* are the most common, and the cow seems to be the most frequent subject. The majority of the cattle which we use for dissecting subjects are affected with tuberculosis, and the cysts are more common in these animals than they are in sound ones. One fine specimen of ovarian cyst was found in a bitch, dead from other cause. In the museum is a beautiful series of cysts in the broad ligament of a sow, in which Dr. T. B. Rogers had pinched off the fimbriated extremities of the Fallopian tubes, some six months previously. *Sebaceous cysts* occurred back of the ears in the horse and in various parts of the dog. "Shoe boils" in the horse and large dogs are frequent examples of cysts. *Dermoid cysts* are of frequent occurrence in the domestic animals. We have one large cyst of the fowl with feathers some three inches in length, and one dermoid cyst from the kidney of a hog.

II. Lipoma.—Fatty tumors were found in four cases from the sides and lips of the dog; in one case on the inside of the thigh, the tumor hung nearly to the ground, while a sixth in the horse closed the eye by completely filling the superorbital space.

III. Fibromas.—These are perhaps the most frequently met with in veterinary practice. They are found in all limits from, the little *soft peripheral fibromas* which are found under the collar and harness, and prove so painful to the horse and annoying to the driver, to the great *hard deep fibroma* which can hang from the lower part of the neck with a weight of fifty pounds. The first mentioned are common and usually due to a badly fitting collar, but the lymphatic,

***Alveolar and Lymphadenoid Sarcoma* appear to be very rare in the domestic animals. *Actinomycosis* is the cause of a large number of tumors, some five cases, affected in the maxillary bones, and one in the tongue, having been sent to our clinic, but as the locality of the growth so materially alters the nature of the tumor, we have not included them in the classification.

heavy crested horse seems more predisposed to its attacks. The next group may be of any size from a walnut to a pumpkin and are usually the result of the irritation of pressure. These are equally common in the mule ; excessively rare in the dog. A third group of hard fibromas are the scirrhous cords which may follow castration.

Fibromas may occur from diathesis. A mule which was first operated upon at the hospital two years ago for multiple fibromas of the off fore-knee, the face, the neck and the body, and had over thirty individual tumors removed, has since been operated upon regularly every few months and has certainly over a hundred tumors removed. The knee had the skin completely dissected from the deeper structures by a honey-comb of fibrous tissue in which were lodged the growths like so many eggs in a basket. They even penetrated into the interspaces between the flexors of the metacarpus.

IV. Papilloma.—Multiple papilloma of the mouth in a dog and the case mentioned above in carcinomas, represent this species. A number of cases in the pastern of the horse and mule are the result of thrush, grease or other irritants. One case at the anus of a mule. One case on the penis of a horse.

V. Adenoma.—Is found most frequently in the mammary glands of the bitch (three cases).

VI. Osteoma.—Osteophytes and exostosis are too frequent in the horse to demand further detail than references to the report of the surgical clinic. A large exostosis on the frontal and parietal bones of a bull caused death by pressure of the parietal bone on the cerebellum and posterior extremity of cerebral lobe.

VII. Chondroma.—One case of chondroma of testicle in a horse.

VIII. Myxoma.—Frequent on nictitans of dog, and in the mucous cysts in the ears of the same animal. One case of myxoma of the choroid plexus in the horse, one side of the older growth being the size of a hazel nut, the other the size of a small egg, completely filling the lateral ventricle.

IX. NEUROMA.—A small painful Neuroma of radial nerve of horse (following the operation of Neurotomy.)

X. RHABDO-MYOMA.—From the left kidney of a hog, congenital.

XI. LYMPHOMA.—A knot of tumors from neck in a hog.*

* Angiome, Lymph-Angiome, had been met with, but was not operated upon. Uterine Myomata and Fibroma do not appear to occur in domestic animals,

